# Shedding the Temporary Teeth

**Published:** 1895-01

**Authors:** J. Taft


### Shedding the Temporary Teeth.

BY J. TAFT.

Read before the Ohio State Dental Society, December 7th, 1894.

In attempting to present some thoughts on this subject it is not with the same confidence that many others would be approached.

It has always been regarded by physiologists, and indeed by all who have made it a subject of study, as one of nature's obscure processes, one that in its full elucidation has escaped the investigations of not only the physician, but the dentist as well, and this, notwithstanding it is his special province to investigate all questions pertaining to odontology.

To all questions pertaining to the teeth the dentist is expected to give special attention, and go farther into the mysteries pertaining to them than others whose studies and investigations necessarily have a broader scope.

The dentist should have as thorough knowledge of all that pertains to the teeth—their development, growth, use, diseases, affections and susceptibilities as possible, in order that he may best secure the welfare of those committed to his care.

This subject of shedding the temporary teeth, is one that in no small degree influences the development and arrangement of the permanent teeth.

It may be well before going further, to note the condition of the child at the time of the development of the teeth, and the purposes they are intended to serve.

The rudiments of the deciduous teeth are formed at an early period of intra-uterine life, and progress on to the perfected state, complete within from three to four years. The teeth unlike other parts of the body are formed complete in a comparatively brief period, and neither increase nor diminish in size. They are then in condition to perform their functions fully.

When the conditions are normal this is the time of life when a change is required in the form of food, from the fluid to the more solid condition; a condition requiring mastication and insalivation. This is a necessary process preliminary to proper digestion.

The jaws as a whole, and especially the parts occupied by the temporary teeth, grow rapidly after the completion—full eruption—of the deciduous teeth, and especially if their work of mastication is well performed. If, however, there is deficiency in this respect, there will be a corresponding slowness in the growth of the jaws.

Where the teeth during infancy and childhood are properly used in mastication, the jaws will have a normal growth, which will usually be shown by the separation of the teeth, especially the six anterior teeth. This separation is far less frequent with the teeth that have been little or not at all used in mastication. The proper use of these teeth is a stimulus to the process of nutrition in the parts about them.

In those cases only, in which the teeth are rightly used, will there be a good, vigorous, nutrient action. Under such circumstances it is not only the jaw, the living structure in which they stand, that receives the benefit, but the teeth that are thus used are better nourished, stronger, and better resist disease.

But, perhaps, of more importance than this, is the active, nutrient supply thus given to the growing permanent teeth, that are as yet enclosed in appropriate cavities in the jaws.

The periosteum of the temporary teeth and of the permanent, so far as their roots may be formed, partake of the same beneficent supply.

The tissue or organ that is to be the active agent in the removal of the roots of the temporary teeth, is also better formed or developed, and will be in better condition for its appropriate function, when the time arrives for its operation.

The temporary teeth are designed in the human organization to have but a brief existence, having an active service of from five to seven years, upon an average.

At the end of this period, they are by a beneficent and *purely* physiological process removed; this is accomplished by the removal of the roots of these teeth. When the process is without interruption or interference the roots are wholly removed, or sufficiently so to permit of the removal of the crown by the ordinary movements of the jaws, the cheeks, lips and tongue.

The manner in which, and the agency by which, the roots of the temporary teeth are moved have afforded ground for various opinions.

The subject has not been much discussed in recent years. The writers of a quarter of a century ago and more gave much more attention to it than those of recent times. Whether this state of affairs has existed because of the difficulties attending its investigation, or because it was supposed or assumed that it had been exhausted, I will not venture to assert. Perhaps it was because subjects more inviting were presented.

It may, however, be said that the process of shedding the teeth is not generally understood, it is therefore a subject that should be further considered.

Various theories have been put forth in the past, as to the manner in which this removal is accomplished.

In the early periods of medical science many supposed that the temporary teeth had no roots. Others advanced the theory that the crowns separated from their roots in the same manner as the horns of a stag fall off, and that from the root the new tooth sprang. Fouchard and Bourdet suggested that the roots of the temporary teeth were removed by the action of a solvent fluid.

Bullow advanced the theory that they were worn away by the advancing (rising), tooth.

Lecluse suggested that when the process of their removal begins their vessels cease to supply the nourishing juices, and that they are broken up by a species of maceration; and Jordan believed it is accomplished by both abrasion and corrosion.

Fox attributes it to the pressure of the crown of the permanent tooth upon the root or roots of the temporary; but he afterwards admits that it frequently occurs without such pressure, and further remarks: "These circumstances seem to prove that the absorption of the fangs of the temporary teeth is an action of nature sometimes independent of pressure; and it is a very singular circumstance, that at a time of life when so great a quantity of ossific matter is poured forth from all the arteries concerned in the formation of bone, in one particular, there should thus be an absorption of this substance taking place."

Bell, rejects the theory of pressure, and attributes the removal to the action of absorbent vessels.

Laforgue observing a fungiform or fleshy substance behind the roots of the temporary tooth, supposed this to be the agent actively engaged in the removal of the temporary teeth.

Bourdet observing essentially the same thing, came to the same conclusion, namely, that a fluid was exhaled from this substance which possessed solvent properties, he gave it the name of the "Absorbing Apparel," and regarded it as the active agent in removing the roots of the temporary teeth.

Delabarre investigated very fully this subject. He corroborates the views of Laforgue, and gives the following description of the manner of the formation and function of this absorbing organ:

"While the crown of the tooth of replacement is in process of formation the exterior membrane of the matrix is simply crossed by blood vessels, but as soon as it is completed the capillaries are then developed in a very peculiar manner, and form a fine tissue. From this tissue the internal membrane instead of continuing to be very delicate, and of a pale red color, increases in thickness and assumes a redder hue. As has been said, it is at the instant in which commences the reaction of the coats of the matrix, that are conveyed from the gum to the neck of the teeth, that the fine vessels enter the tissue, thus composing a body of carneous, flesh-like appearance. It is therefore the dental matrix itself, that without being dilated to serve as a protecting envelope, is contracted to form not only its bud-like body, which we find below the temporary tooth when it is shed, but also a carneous mass by which the whole is surrounded."

He then asks, "Is there an absorbing fluid that acts chemically on the surrounding parts, or do the absorbents, without any intermedial, destroy everything that would obstruct the advance of the permanent tooth?"

In reply to this he says, "Not possessing positive truth to guide me in the decision of this question, and finding those of others of little importance, I shall not attempt to give answer."

These views would seem to approximate more nearly a correct solution of this process than those before referred to, though there are some features of this process which all writers state have not been clearly understood. And while there are questions not now fully comprehended, there are certain points, we think, which may be regarded as settled.

And first we have no hesitancy in stating that the process of shedding the temporary teeth is a purely physiological one; though it has been suggested by some writers, of later date than those referred to, that inflammation in a more or less marked degree, is present during the accomplishment of this work. This we cannot accept as yet proven; and may state that in a great majority of cases there is no indication whatever of inflammation, or even special irritation. It is true that oftentimes local disease of more or less marked character is concurrent with shedding the teeth; but that it occurs as a necessary result of the removal of the roots of the temporary teeth, we think, there is no evidence.

In the plan of the human organism the temporary teeth are to be displaced to make way for the permanent; and, it would seem to be unreasonable that diseased action, even in the mildest form, should be called in to aid in the accomplishment of this work. Disease never occurs without some violation of physiological law.

Again, it is admitted by all intelligent observers that this work is accomplished by the solution of the material to be removed; and this cannot be accomplished without the intervention of a solvent. The debris from the wasting structure is absorbed and carried away through the proper eliminating channels. In order to be absorbed, and thus disposed of, the material operated upon must be in absolute solution.

Third, the fact that is clearly shown by intelligent observation is, that both the organic and inorganic constituents of the cement and dentine are simultaneously removed. An examination of the wasting surface does not present a softened condition, as is shown in decay of the teeth, and as would be shown here if the inorganic material was first removed; nor is there a chalky condition of the wasting surface, as would be shown if the organic constituents were first removed.

We are, therefore, forced to the conclusion that the agent concerned in the removal is one having an equal action, so far as its solvent power is concerned, upon both the organic and inorganic constituents.

There are certain uniform features in the accomplishment of this process that indicate quite clearly the fact that it is governed by true physiological law. This is shown by its operation at about a uniformly given time, and by its operation upon certain pairs of teeth.

This manifest law rules out inflammation or any diseased action as a necessary factor in the process.

The removal of the roots of the temporary teeth has definite periods for its accomplishment; which is always carried out according to the plan, when there is no diseased or abnormal condition present. Neither inflammation nor disease of any kind is uniform in its attack or mode of operation.

The removal of the roots of these teeth is accomplished in nearly all cases without any indication of disease, either in appearance or sensibility. Hence the conclusion is well nigh forced upon us, that the removal of the roots of the temporary teeth is by a purely physiological law; and that disease in none of its manifestations is a factor, even in the slightest degree, in its accomplishment.

A fourth point, recognized by all close observers, is that the surface of the root being acted on is always in opposition to the corresponding and approaching permanent tooth.

Fifth, there is always found an intervening soft, highly vascular tissue between the permanent crown and the temporary root during the time of the removal of the latter.

Sixth, these hard surfaces are never found in contact when in a normal condition.

Seventh, the root of the temporary tooth is not removed when the crown of the permanent is absent, or its growth arrested, or is situated at considerable distance from the root.

Eighth, the root is not removed by this absorbing process, in the absence, from whatever cause, of this intervening vascular body or absorbent organ.

This organ is in some cases, though rare, never developed. In other cases it is destroyed by disease. In either case the root is not absorbed; and this even though the permanent tooth may grow and be erupted, when usually the permanent tooth is made to take a wrong position.

[Discussion of the above paper will appear in a subsequent number of THE DENTAL REGISTER.—ED.]

